# Medication Adherence among Patients with Chronic Diseases in Saudi Arabia

**DOI:** 10.3390/ijerph191610053

**Published:** 2022-08-15

**Authors:** Khulud Alosaimi, Hassan Alwafi, Yosra Alhindi, Alaa Falemban, Asim Alshanberi, Nahla Ayoub, Safaa Alsanosi

**Affiliations:** 1 Department of Pharmacology and Toxicology, Faculty of Medicine, Umm Al-Qura University, Makkah 24382, Saudi Arabia; 2 Pharmacy Department, King Faisal Medical Complex, Taif 26514, Saudi Arabia; 3 Saudi Toxicology Society, Umm Al-Qura University, Makkah 24382, Saudi Arabia; 4 Department of Community Medicine, Faculty of Medicine, Umm Al-Qura University, Makkah 24382, Saudi Arabia; 5 BHF Glasgow Cardiovascular Research Centre, Institute of Cardiovascular and Medical Sciences, University of Glasgow, Glasgow G12 8QQ, UK

**Keywords:** medical prescribing, medication adherence, chronic diseases, medication non-adherence

## Abstract

Introduction: The management of chronic illnesses commonly includes a long-term pharmacological approach. Although these medications effectively control disease, their full benefits are often not realized because approximately 50% of patients do not take their medications as prescribed. Medication adherence has become a big concern to clinicians and healthcare systems in Saudi Arabia and worldwide because of growing evidence associating nonadherence with adverse outcomes and higher costs of care. Despite it being a well-recognized problem, few studies have investigated medication adherence in Saudi Arabia. Therefore, this study aims to gain a better perspective on medication adherence among patients with chronic diseases in Saudi Arabia. Method: A questionnaire-based cross-sectional study was conducted among patients with chronic diseases in the Makkah region, Saudi Arabia, from 1 May to 31 July 2021. Patients aged 18 years and above who were taking prescribed or over-the-counter medications were included. Descriptive statistics were used to describe the participants’ characteristics, and categorical variables were reported as frequencies and percentages. A Chi-square test was used to test the relations between variables. Results: In total, 239 participants were included in the study. Females represented 62% of the participants. In terms of the history of chronic diseases, 44% had hypertension, 40% had diabetes mellitus, 21% had heart diseases and 9% had asthma. Nearly half (49%) of participants did not follow up regularly with a primary healthcare center and 42% said that they had forgotten to take their medications in the past. However, most of the participants (78%) stated that they took their medicine as instructed by their doctor or pharmacist, and 61% took their medications on time. The majority of participants (85%) said that the pharmacist explained the method of using the medications and the instructions for use, while 30% thought that the medications they took were too much. In regard to the reasons for medication nonadherence, having no specific reasons for medication nonadherence was the most common cause for nonadherence in our study. The relationship between patients taking medications as instructed by a healthcare provider (the doctor or pharmacist) and the healthcare provider giving clear instructions to patients about medication use was significant (*p* < 0.001). Conclusions: Failure to adhere is a significant problem that not only affects the patient but also the healthcare system. Additional research is needed to monitor medication adherence and identify factors contributing to this problem to provide successful strategies to improve medication adherence in Saudi Arabia.

## 1. Introduction

The increase in life expectancy and aging have influenced the use of life-long medications in chronic disease management. However, the full benefits of these medications are often not recognised, as almost half of patients do not take their medications as prescribed [1]. According to the WHO, adherence is defined as “the extent to which the persons’ behaviour (including medication-taking) corresponds with agreed recommendations from a healthcare provider” [2]. 

Medication adherence has become a big concern in healthcare systems around the world mainly for two reasons: (1) There is no general agreement on the ideal adherence percentage (some studies consider over 80% satisfactory and others require a minimum of 95% adherence). (2) Measuring medication adherence through the use of different interventions for improvement is up now, and uncommon in daily clinical practice [3,4,5]. Clinical studies have shown that medication adherence is much higher in patients with acute cases compared to those with chronic disease [6,7]. Some chronic conditions, such as diabetes mellitus, hypertension and depression have the highest rate of medication nonadherence [8,9,10].

In general, methods of adherence measurement are divided into two types: direct and indirect [11]. Direct methods include direct patient observation, drug level assays, and biomarkers. Indirect methods include self-reporting (such as questionnaires), electronic monitoring devices, pharmacy refill rates, and pill counts [11]. The most used indirect methods include patient self-reports, pill counts, and pharmacy refills [12]. 

In the Saudi healthcare system, most chronic diseases are primarily managed within primary care centres. The physician “signs” the prescription electronically and the staff then sends it directly to the patient’s preferred pharmacy [13]. Studies have shown that medication adherence is considerably low among chronic disease patients in Saudi Arabia [13,14]. To improve adherence, prevent negative outcomes and reduce the burden on secondary care centres, an assessment of medication adherence and the factors that affect it within primary care is required [8].

Medication adherence has become a significant concern for clinicians and healthcare systems in Saudi Arabia and around the world, as non-adherence is associated with adverse outcomes and higher costs of care [6,11]. Still, few studies have investigated medication adherence in Saudi Arabia specifically. Therefore, this study aims to gain a better perspective on medication adherence among patients with chronic diseases in the Makkah region, Saudi Arabia.

## 2. Materials and Method

### 2.1. Ethical Approval

The study was approved by the Biomedical Research Ethics Committee, Faculty of Medicine, Umm Al-Qura University, Makkah, Saudi Arabia. Approval number: HAPO-02-K-012-2021-04-672, under the Declaration of Helsinki.

### 2.2. Study Design

A cross-sectional study was conducted among patients with chronic diseases in Saudi Arabia. They were randomly approached by sending them the electronic questionnaire over the three months from 1 May to 31 July 2021. The purpose of the research was explained to participants in the questionnaire. They were also informed that participation was voluntary.

### 2.3. Questionnaire Tool

The questionnaire was adapted from a previous study by Prabahar K et al. [14]. Experts provided their feedback and opinions for improving the questionnaire, and their suggestions were incorporated into the final questionnaire, which contained 15 questions and was designed using online cloud-based questionnaire development software (Google Forms). The questionnaire was designed in English and translated into Arabic, the local spoken language, by proficient speakers of both languages, and revised to suit the general population. It was divided into three main parts: The first part included sociodemographic information. The second part was about the participants’ medication adherence and their reasons for medication nonadherence. The third part was about medical prescription numbers and instructions, as shown in Table 1**.**

### 2.4. Study Populations (Inclusion/Exclusion Criteria)

The selection criteria included adults (men and non-pregnant women) above 18 years of age with chronic diseases who were taking medications both prescribed and over the counter and attending primary care service in the Makkah region, Saudi Arabia. Exclusion criteria: inability to provide informed consent, pregnancy, concomitant serious medical, or surgical condition requiring hospitalization. 

### 2.5. Sample Size and data Collection

The sample size was calculated using Slovin’s formula, with a population size of 385 patients with chronic diseases in Saudi Arabia from a recently published study by Kurdi S et al. 2021, with a confidence interval (CI) of 95% and a margin of error of 5% [14]. Social media channels were used to distribute the questionnaire. All responses to the questionnaire were downloaded from the Google Forms website and held on a secure server. We received a complete case analysis of the answers provided by respondents who completed all 15 questions from the three-part survey. Participants who provided incomplete responses to the questionnaire were excluded. The data were collected from the spreadsheets provided by Google Forms and transferred to Microsoft Excel.

### 2.6. Statistical Analysis

Data were analysed using SPSS version 23.0 (SPSS Inc., Chicago, IL, USA) and we presented categorical variables as frequency and percentage. We used the Pearson Chi-square test to measure any differences. A *p*-value of <0.05 was considered statistically significant.

## 3. Results

A total of 300 questionnaires were collected, of which 61 were excluded because of incomplete responses, giving a response rate of 80%. In total, 239 questionnaires were included in this study. Females represented 62% of the participants included in the study, as shown in Table 2. In terms of age groups, participants belonging to the 50-years-or-older group comprised the highest percentage (48%), followed by the 30-to-49-year-old group (34%) and lastly the 18-to-29-year-old group (18%). While almost half of the participants (50%) had received an undergraduate education, the other half (50%) had received a basic education (including elementary, intermediate, and high school education). Regarding the history of chronic diseases, 44% had hypertension, 40% had DM, 9% had asthma, 21% had heart diseases, and 23% had other diseases such as thyroid diseases, psychological diseases and rheumatic diseases.

As shown in Table 3, nearly 49% of participants did not follow up regularly with a primary healthcare centre and 42% said that they had forgotten to take their medications in the past. However, most of the participants (78%) stated that they took their medicine as instructed by their doctor or pharmacist and 61% took their medications on time. The majority of participants (85%) said that the pharmacist explained the method of using the medications and the instructions for use, while 30% thought that the medications they took were too much.

In regard to the reasons for medication nonadherence, as shown in Table 4, only 5% of participants said that they did not adhere to their medications because they did not understand the instructions, while 15% experienced side effects or were afraid of getting used to the drug. While almost half of the participants (48%) said they had forgotten to take their medications, 31% were too busy to take them. Interestingly, having no specific reason for medication nonadherence was the most common cause for nonadherence in our study.

As shown in Supplementary Materials Table S1, over half of the participants (55%) took one to three medications, followed by 30% who took four to six medications, 11% who took seven to nine medications, and only nine participants said they took more than ten medications.

As shown in Table 5, the Chi-square test to compare whether there was a relationship between patients taking medications as instructed by a healthcare provider (the doctor or pharmacist) and the healthcare provider giving clear instructions to patients about medication use showed that there was a dependent relationship (*p* < 0.001). However, taking medications as instructed by the doctor or pharmacist was not related to the number of medications taken per day (*p* = 0.307), as shown in Supplementary Materials Table S2.

## 4. Discussion

This study aimed to achieve a better view of drug adherence and involvement, especially in patients with chronic diseases, in the Makkah region, Saudi Arabia. Adherence to drugs and regimen protocols is essential for therapeutic achievement. Consequently, failure to adhere is a major problem that affects patients and the healthcare system [15]. Moreover, it leads to the considerable worsening of disease and death, along with high health care costs [4]. Females represented the higher gender percentage (62%) in the current study. Clinical studies have found that women report more health problems than men in both developing and developed countries with regard to their economic status [16]. Moreover, the percentage of medication nonadherence is higher in women compared to men who use chronic medications, and they are unlikely to obtain the pharmacological treatment and monitoring recommended by clinical guidelines [17].

There are different types of medication nonadherence. The first is known as nonfulfillment adherence, in which healthcare providers write a prescription but the prescription is never filled or the medication started [18]. However, in this study, most of the reasons for nonadherence came under the second type of nonadherence, known as non-persistence adherence, in which patients choose to stop following up with PHC centres (e.g., almost half of our participants (49%) stopped following up with PHC centres) or stop taking their medications after starting them without consulting their doctors or pharmacist [1]. Non-persistence is mainly unintentional and occurs when patients and their healthcare providers miscommunicate about the management plan [19]. For instance, about 28% of our participants stated that when they felt better, they sometimes stopped taking medications, and 11% increased the dose without consulting their healthcare provider.

In the current study, the most common reason for medication nonadherence was having a lot of medications (86% of participants). Taking a lot of medications is known as polypharmacy. However, there is a wide range of definitions for polypharmacy, ranging from two medications to ten or more medications. The most commonly used definition of polypharmacy is using five or more medications [20]. In our study, 30% of participants took four to six medications, which comes under the most used definition for polypharmacy. Several studies have shown that polypharmacy plays a significant role in medication nonadherence, and by lowering the number of prescribed medications and simplifying the management plan the patient becomes more adherent to their chronic medications [21,22,23,24]. Taking medication as prescribed plays an important role in controlling chronic conditions, managing temporary conditions, and general long-term health and wellbeing. The connection with the healthcare provider is a crucial part of medication adherence [15,25]. In our study, 85% of participants said that their healthcare provider gave them clear instructions about their medication use. Effective conversation improves the patient–health practitioner relationship, decreases both medication errors and nonadherence, and improves the general medical results and patients’ physical and intellectual fitness associated with their diseases [26].

In this study, 42% of participants said that they had forgotten to take their medications in the past. Forgetfulness is an important contributor to nonadherence, especially with chronic disease medications, and could be addressed with medication reminder devices [27]. Different medication reminder applications on smartphones and electronic watches have been proposed and used by patients. However, using straightforward and clear medical instructions seems to be the simplest approach [28]. In our study, we found that the relationship between taking medications as instructed by a healthcare provider and having clear medical instructions was significant (*p* < 0.001). This highlights the importance of spending more time with the patients to give clear medical instructions, such as dosing instructions, and answering patients’ enquiries [29]. Consequently, developing a standard procedure to provide complete, clear, and simple medical instructions to patients and continuous education for physicians and pharmacists on the proper communication of medication instructions to patients are recommended [30].

A few limitations should be considered when reading the results of this study. First, our study may not be representative because of the study design (online survey), as it might not capture the responses from other patients who do not use social media. Second, the study did not determine classes or subclasses of medications associated with medication nonadherence. Despite these limitations, the present study conducted among patients with chronic diseases in Saudi Arabia will add to our knowledge in this area as we still have a limited number of studies addressing medication nonadherence among chronic patients in general. Moreover, we included patients who were taking both prescribed and over-the-counter medications since previous studies have focused on medication nonadherence in certain drug classes or diseases.

## 5. Conclusions

Failure to adhere is a significant problem that affects not only the patient but also the healthcare system. Barriers to adherence include patient, provider, and health system factors, with connections among them. Determining the barriers for each patient and implementing appropriate techniques to overcome them will be needed to improve medication adherence. Patient education and motivation play significant roles in improving adherence. Healthcare professionals should find practical strategies to enhance medication adherence in their practices, which will enhance therapeutic outcomes. In conclusion, additional research is needed to monitor medication adherence and identify factors contributing to this problem to provide successful strategies to improve medication adherence in Saudi Arabia.

## Figures and Tables

**Table 1:** The questionnaire.

You are invited to participate in a research study about medication adherence in chronic disease patients. The aim of this research study is to gain a better perspective on medication adherence among patients with chronic diseases in Makkah region, Saudi Arabia. This research is conducted by the Department of Pharmacology and Toxicology, Faculty of Medicine, Umm Al-Qura University, Makkah, Saudi Arabia. Participation is voluntary and anonymous. If you agree to participate in this study, you can start answering the questionnaire. Participation may not benefit you directly, but it will help us learn about medication adherence in Saudi Arabia. Thank you for your time and collaboration.			
1 Sociodemographic			
Age:			18–29 30–49 50 or older
Sex:			Male Female
Educational level:			Basic Undergraduate Postgraduate
What diseases do you suffer from (you can choose more than one option, if any)?			Hypertension Diabetes Heart diseases Asthma Others______Specify
2 Medication adherence			
	Yes	No	Sometimes
Did you follow regularly with a primary health care center?			
Do you take your medications on time?			
Have you ever stopped taking medicines for any reason?			
Have you ever increased the dose more than required?			
When you feel better, do you sometimes stop taking medications?			
Have you forgotten to take your medications before?			
Do you adhere to taking your medicine as instructed by your doctor or pharmacist?			
If you are “Not adhered” to taking medications correctly, what are the reasons for that? (You can choose more than one option, if available) Not understanding the instructions The occurrence of side effects Fear of getting used to the drug Lack of trust in the health care provider (Doctor or Pharmacist) A lot of medications Long duration of treatment Forget to take the medication Busy to take the medication Symptoms disappeared or felt better Doubting the effectiveness of the medication No specific reason			
3 Medical prescriptions			
How many types of medications do you take per day? 1–3 4–6 7–9 10 or more			
	Yes	No	Sometimes
Do you think that the medications you take too much?			
Did the doctor or pharmacist gave you clear instructions about the medications uses?			

**Table 2:** Demographic characteristics of participants.

Demographics	Numbers (%) N = 239
Age	
18–29	44 (18%)
30–49	80 (33%)
50 or more	115 (48%)
Gender	
Female	149 (62%)
Male	90 (38%)
Educational level	
Basic	120 (50%)
Undergraduate	119 (50%)
History of chronic diseases	
Hypertension	106 (44%)
Diabetes mellitus	96 (40%)
Asthma	21 (9%)
Heart diseases	51 (21%)
Others	56 (23%)

**Table 3:** Level of medication adherence among the participants.

	Yes	No	Sometimes	Total
Do you follow up regularly with a primary health care center?	75 31.4%	118 49.4%	46 19.2%	239 100%
Do you take your medications on time?	145 60.7%	21 8.8%	73 30.5%	239 100%
Have you ever stopped taking medications for any reason?	85 35.6%	112 46.9%	42 17.6%	239 100%
When you feel better, do you sometimes stop taking medications?	67 28%	132 55.2%	40 16.7%	239 100%
Have you ever increased the dose more than required?	25 10.5%	192 80.3%	22 9.2%	239 100%
Have you ever forgotten to take your medications?	101 42.3%	84 35.1%	54 22.6%	239 100%
Do you take your medications as instructed by the doctor or pharmacist?	186 77.8%	6 2.5%	47 19.7%	239 100%
Did the doctor or pharmacist give you clear instructions about medication use?	203 84.9%	19 7.9%	17 7.1%	239 100%
Do you think that the medications you take are too much?	72 30.1%	103 43.1%	64 26.8%	239 100%

**Table 4:** Reasons for medication nonadherence.

	Yes	No	Total
Not understanding the instructions	13 5.4%	226 94.6%	239 100%
The occurrence of side effects	35 14.65	204 85.4%	239 100%
Fear of getting used to the drug	37 15.5%	202 84.5%	239 100%
Lack of trust in the health care provider (doctor or pharmacist)	6 2.5%	233 2.5%	239 100%
A lot of medications	205 85.8%	34 14.2%	239 100%
Long duration of treatment	43 18.0%	196 82.0%	239 100%
Forget to take the medication	114 47.7%	125 52.3%	239 100%
Too busy to take the medication	75 31.4%	164 68.6%	239 100%
Symptoms disappeared or felt better	21 8.8%	218 91.2%	239 100%
Doubting the effectiveness of the medication	10 4.2%	229 95.8%	239 100%
No specific reason	189 77.8%	53 22.2%	239 100%

**Table 5:** Relationship between (1) taking medications as instructed by a healthcare provider and (2) the healthcare provider giving clear instructions about the medications used. Statistical significance was determined at a *p*-value of <0.05.

			Do You Take Your Medications as Instructed by the Doctor or Pharmacist?			Total	
			No	Yes	Sometimes		
Did the doctor or pharmacist give you clear instructions about medication use?	No		5	8	6	19	239
	Yes		1	167	35	203	
	Sometimes		0	11	6	17	
		Asymptotic Significance (2-sided)					
Pearson Chi-square		<0.001					
Likelihood Ratio		<0.001					

## Data Availability

Data available upon request.

## Supplementary Materials

The following supporting information can be downloaded at: https://www.mdpi.com/article/10.3390/ijerph191610053/s1.
