# Supplementary material for: Medication Adherence among Patients with Chronic Diseases in Saudi Arabia
Source: Int J Environ Res Public Health. 2022 Aug 15;19(16):10053. doi: 10.3390/ijerph191610053 (PMC9408114; doi:10.3390/ijerph191610053)
Supplement: Supplementary file 1 [file ijerph-19-10053-s001.zip › ijerph-1798383-supplementary.pdf]

Supplementary Materials

Online supplement

Table S1. Number of medications taken per day.

		Frequency	Percent	Valid Percent	Cumulative Percent
**Medications taken per day**	1 to 3	**132**	55.2	55.2	55.2
	4 to 6	72	30.1	30.1	85.4
	7 to 9	26	10.9	10.9	96.2
	10 and more	9	3.8	3.8	100.0
	Total	239	100.0	100.0	

Table S2. Relationship between [1] Taking medications as instructed by the doctor or pharmacist [2] Number of medications taken per day. Statistical significance was determined at a P-value of <0.05.

		**Do you take your medications as instructed by the doctor or pharmacist?**			Total	
		**No**	Yes	Sometimes		
**Number of medications taken per day**	**1 to 3**	**4**	108	20	132	239
	4 to 6	2	50	20	72	
	7 to 9	0	20	6	26	
	10 and more	0	8	1	9	

	**Asymptotic Significance (2-sided)**
Pearson Chi-Square	.383
Likelihood Ratio	.307
